# Supplementary material for: Ahf-Caltide, a Novel Polypeptide Derived from Calpastatin, Protects against Oxidative Stress Injury by Stabilizing the Expression of CaV1.2 Calcium Channel
Source: Int J Mol Sci. 2023 Oct 29;24(21):15729. doi: 10.3390/ijms242115729 (PMC10648788; doi:10.3390/ijms242115729)
Supplement: Supplementary file 1 [file ijms-24-15729-s001.zip › ijms-2664505-supplementary.pdf]

Article

Ahf-Caltide, a Novel Polypeptide Derived from Calpastatin, Protects against Oxidative Stress Injury by Stabilizing the Expression of Cav1.2 Calcium Channel

Yingchun Xue [1,†], Shi Zhou [1,†], Ling Yan [1], Yuelin Li [1], Xingrong Xu [1], Xianghui Wang [1], Etsuko Minobe [2], Masaki Kameyama [2], Liying Hao [1,*] and Huiyuan Hu [1,*]

1. Department of Pharmaceutical Toxicology, School of Pharmacy, China Medical University, Shenyang 110122, China
2. Department of Physiology, Graduate School of Medical and Dental Sciences, Kagoshima University, Kagoshima 890-8544, Japan

* Correspondence: lyhao@cmu.edu.cn (L.H.); hyhu@cmu.edu.cn (H.H.); Tel.: +86-133-9056-9003 (L.H.); +86-189-0091-1978 (H.H.)

† Co-first author: These authors contributed equally to this work.

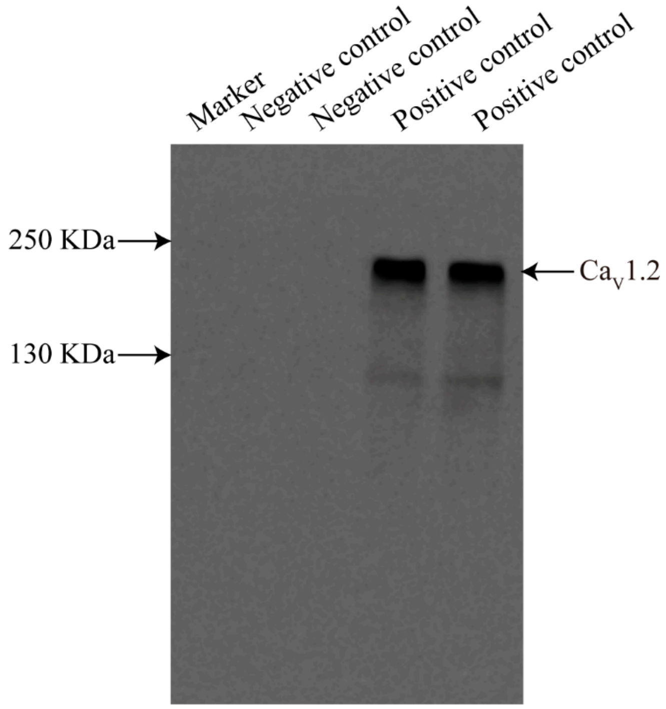

Supplemental Figure S1. The specificity validation of Cav1.2 antibody by Western blot. The bovine serum albumin was used as negative control and the left ventricle of rat was used as positive control.
